# Perioperative risk factors for recovery room delirium after elective non-cardiovascular surgery under general anaesthesia

**DOI:** 10.1186/s13741-020-00174-0

**Published:** 2021-02-03

**Authors:** Jiayi Wu, Shaojie Gao, Shuang Zhang, Yao Yu, Shangkun Liu, Zhiguo Zhang, Wei Mei

**Affiliations:** 1 Department of Anesthesiology, Tongji Hospital, Tongji Medical College, Huazhong University of Science and Technology, 1095 Jiefang Avenue, Wuhan, 430030 China; 2 School of Medicine and Health Management, Tongji Medical College, Huazhong University of Science and Technology, 13 Hangkong Road, Wuhan, 430030 China

**Keywords:** General anaesthesia, Delirium, Postoperative recovery, Risk factors, Non-cardiac surgery

## Abstract

**Background:**

Although postoperative delirium is a frequent complication of surgery, little is known about risk factors for delirium occurring in the post-anaesthesia care unit (PACU). The aim of this study was to determine pre- and intraoperative risk factors for the development of recovery room delirium (RRD) in patients undergoing elective non-cardiovascular surgery.

**Methods:**

RRD was diagnosed according to the Confusion Assessment Method for the Intensive Care Unit (CAM-ICU). We collected perioperative data in 228 patients undergoing elective non-cardiovascular surgery under general anaesthesia and performed univariate and multivariate logistic regression to identify risk factors related to RRD. PACU and postoperative events were recorded to assess the outcome of RRD.

**Results:**

Fifty-seven patients (25%) developed RRD. On multivariate analysis, maintenance of anaesthesia with inhalation anaesthetic agents (OR = 6.294, 95% CI 1.4–28.8, corrected *p* = 0.03), malignant primary disease (OR = 3.464, 95% CI = 1.396–8.592, corrected *p* = 0.018), American Society of Anaesthesiologists Physical Status (ASA-PS) III–V (OR = 3.389, 95% CI = 1.401–8.201, corrected *p* = 0.018), elevated serum total or direct bilirubin (OR = 2.535, 95% CI = 1.006–6.388, corrected *p* = 0.049), and invasive surgery (OR = 2.431, 95% CI = 1.103–5.357, corrected *p* = 0.035) were identified as independent risk factors for RRD. RRD was associated with higher healthcare costs (31,428 yuan [17,872–43,674] versus 16,555 yuan [12,618–27,788], corrected *p* = 0.002), a longer median hospital stay (17 days [12–23.5] versus 11 days [9–17], corrected *p* = 0.002), and a longer postoperative stay (11 days [7–15] versus 7 days [5–10], corrected *p* = 0.002]).

**Conclusions:**

Identifying patients at high odds for RRD preoperatively would enable the formation of more timely postoperative delirium management programmes.

## Introduction

Delirium is an acute brain organ dysfunction characterized by changes in level of consciousness, inattention, and disorganized thinking. Postoperative delirium, one of the most frequently encountered complications observed postoperatively, is a transient mental dysfunction that can result in increased morbidity, delayed functional recovery, and prolonged hospital stay (Lepouse et al. 2006). In clinical practice, it is common to classify delirium as: (1) hypoactive subtype, characterized by reduced alertness, sedation, and reduction of motor activity; (2) hyperactive form, associated with hyper-vigilance, psychotic features (e.g. hallucinations and delusions) and agitation (Fields et al. 2018); and (3) a more prevalent, mixed subtype with overlapping features of the previous two forms. Risk factors related to postoperative delirium have been identified previously (Inouye and Charpentier 1996; Marcantonio et al. 1994). Inouye et al. reported that a risk factor intervention strategy significantly reduced the number and duration of delirium episodes (Inouye et al. 1999). Despite the importance of early recognition and timely management of delirium, recovery room delirium (RRD) in the post-anaesthesia care unit (PACU) has not been extensively investigated (Card et al. 2015; Fields et al. 2018; Lepouse et al. 2006; Radtke et al. 2008; Sharma et al. 2005). As a result of different diagnostic criteria and definitions of delirium, the incidence rate of RRD ranges from 3 to 21.1% (Juliebo et al. 2009; Lepouse et al. 2006; Radtke et al. 2008). Previous studies have mainly considered the hyperactive subtype of postoperative delirium (agitation) and not the hypoactive subtype (Lepouse et al. 2006). Several scales have recently been validated for assessing delirium in the PACU setting (Radtke et al. 2008). User-friendly and reliable tools, such as the Confusion Assessment Method for the Intensive Care Unit (CAM-ICU), allow the clinician to identify both hyperactive and hypoactive delirium in the postoperative setting (Card et al. 2015; Ely et al. 2001). CAM-ICU was validated for delirium assessment for mechanically ventilated critically ill patients (Ely et al. 2001) or non-intubated patients (Van Rompaey et al. 2008) in various settings such as surgical ICU (Guenther et al. 2010), emergency department (Han et al. 2010), mixed intensive care unit (van Eijk et al. 2009), surgical and trauma intensive care unit (Pandharipande et al. 2008), trauma unit (Soja et al. 2008), as well as PACU setting (Card et al. 2015). The CAM-ICU has a higher specificity than sensitivity for delirium when used in the PACU (Neufeld et al. 2013). Identifying patients at high odds for RRD preoperatively would enable the formation of more timely postoperative delirium management programmes (Munk et al. 2016). In this prospective study, we used the CAM-ICU to investigate the proportion of and risk factors associated with RRD in PACU after elective non-cardiovascular surgery under general anaesthesia. We also investigated which postoperative factors occurred at a significantly higher proportion in patients who developed recovery room delirium.

## Methods

### Patients

This observational study was reviewed and approved by the Hospital Institutional Review Board of Tongji Hospital, Huazhong University of Science and Technology, Wuhan, China, and registered with Clinical Trials (NCT00991913). All patients gave written informed consent before induction of anaesthesia. Patients older than 18 years, who were admitted to the PACU after elective non-cardiovascular surgery under general anaesthesia during regular working hours, 9:00 am to 5:00 pm, were screened on eight randomly selected working days in June 2010. Patients were not included consecutively, due to a lack of personnel capacity for delirium evaluation in the busy PACU setting, but were representative of the patient population at the Tongji Hospital of the Huazhong University of Science and Technology, Wuhan, with respect to age, comorbidity, and surgical procedures. The 12-bed PACU is located next to the operating rooms in Tongji Hospital, a general university teaching hospital. Two well-trained researchers in the PACU were responsible for patient evaluation. The anaesthetist in charge was responsible for patient discharge. Transition from PACU to surgical ward was considered safe when patient had achieved a Modified Aldrete Score of 9 (Aldrete 1995).

All patients received 1 to 2 mg midazolam soon after arriving at the operating room. General anaesthesia was induced with propofol or etomidate in combination with fentanyl or remifentanil, followed by neuromuscular block with either vecuronium or rocuronium to facilitate endotracheal intubation. Anaesthesia was maintained by total intravenous anaesthesia (TIVA) using propofol or inhalation anaesthetics, either isoflurane or sevoflurane. The anaesthetist in charge was free to use opioid analgesics and muscle relaxants as needed. All patients were extubated in operation theatre at the end of surgery. The anaesthesiologist responsible for the patient’s care was not aware of the inclusion of the patient in the study before or during surgery.

Exclusion criteria were age < 18 years, refusal to sign consent form, operation under regional anaesthesia, history of substance dependence (including opioid, alcohol, or nicotine), neurosurgical procedure, history of primary neurologic disease, and admission to PACU with stays of less than 10 min.

### Outcome

Our primary outcome was the presence of delirium in PACU determined by CAM-ICU (Ely et al. 2001). The CAM-ICU Simplified Chinese version was obtained from http://www.icudelirium.org/. Ten minutes after the arrival of patients in the PACU, the patients were assessed with Richmond Agitation-Sedation Scale (RASS). If RASS was < − 2, then the patient was assessed again after 5 min. If RASS was ≥ − 2 or more, trained research assistants assessed delirium by the CAM-ICU.

In order to make a reliable diagnosis of recovery room delirium in the very busy PACU setting, we have chosen CAM-ICU based on the following considerations: (1) CAM-ICU flowsheet was proved to be the most reliable instrument for delirium assessment in many settings under various cultures including surgical ICU settings in Germany (Guenther et al. 2010; Luetz et al. 2010), a Swedish ICU setting (Larsson et al. 2007), a mixed medical-surgical ICU setting in the Netherlands (Spronk et al. 2009), ICU setting in Chinese populations (Chuang et al. 2007), and PACU setting (Card et al. 2015); (2) CAM-ICU flowsheet allows a quick assessment that needs only 50 s (interquartile range, 40–120 s) in patients with delirium vs 45 s (interquartile range, 40–75 s) in those without delirium to complete assessments (Guenther et al. 2010), which would be a great advantage for use of CAM-ICU in the busy settings such as PACU; (3) PACU settings are similar with surgical ICU settings in our hospital, and Chinese version of CAM-ICU was tested in a prior study in Chinese population shown good validity and reliability (Chuang et al. 2007).

The CAM-ICU evaluates the following four ‘features’ of delirium: (i) an acute change in mental status or fluctuation in the level of consciousness over the prior 24 h, (ii) inattention, (iii) disorganized thinking, and (iv) an altered level of consciousness. The CAM-ICU has a higher specificity than sensitivity for delirium when used in the PACU (Neufeld et al. 2013). Thus, we expected fewer false positives than false negatives, thereby taking a conservative approach to the determination of the proportion of delirium in our cohort. The CAM-ICU was administered in the PACU by two research assistants who each received one-on-one training plus quality assurance review of 10 independent assessments before the start of the study by a CAM-ICU expert at our institution (WM). The k-statistic for agreement between the expert and each of the assessors was 1.0 indicating perfect agreement.

### Candidate predictors

Predictors in the present study were selected according to their clinical importance and based on the results of previous studies. Demographic and pre- and intraoperative variables, including age, gender, weight, American Society of Anaesthesiologists Physical Status (ASA-PS) (III–V versus I–II), preoperative haemogram (white blood cell count, haemoglobin, and haematocrit), preoperative serum biochemistry (sodium, potassium, chloride, calcium, creatinine, blood urea nitrogen (BUN), cholesterol, uric acid, glucose, total bilirubin, direct bilirubin, albumin, and total protein), preoperative routine hepatic enzymes (alanine transaminase and aspartate transaminase), diagnosis of primary disease (malignant versus benign), type of surgery (invasive versus mini-invasive), location of surgery (head and neck, intrathoracic, intra-abdominal, urogenital, musculoskeletal and spinal, or peripheral), maintenance of anaesthesia (inhalation anaesthetic versus TIVA), preoperative and intraoperative haemodynamic parameters (maximal and minimal heart rate, maximal and minimal systolic/diastolic blood pressure), preoperative and intraoperative oxygen saturation, intraoperative fluid application, intraoperative loss of body fluid (including blood loss, urinary production, and any other obvious fluid loss), duration of surgery (≥ 2 h versus < 2 h), and perioperative hospital length of stay (LOS) were evaluated by viewing patient data records. We categorised the laboratory values as normal or abnormal based on the normal values of the clinical laboratory at Tongji Hospital. We performed univariate and multivariate analyses to identify independent risk factors for delirium.

We also recorded PACU and postoperative events, including maximal heart rate; maximal and minimal systolic blood pressure (SBP) in PACU; mean oxygen saturation in PACU; PACU-, postoperative-, and total hospital-LOS; total healthcare costs and healthcare costs per day during hospital stay, to assess the relationship between RRD on these variables.

### Statistical analysis

Descriptive statistics were computed for all study variables. We used Kolmogorov-Smirnov and Shapiro-Wilk tests and normal-quantile plots to determine whether continuous variables were normally distributed. Because most variables had a non-normal or asymmetric distribution, we have reported results as median [25–75% percentiles] rather than mean ± SD and used nonparametric statistical tests. Differences between the two patient groups (delirium versus no delirium) were tested by univariate and multivariate methods. We conducted Chi-square tests (Fisher’s exact test) or Mann-Whitney *U* tests for each variable to reduce the number of variables included in the multivariate model. *P* values in univariate analysis were not adjusted. In order to reduce the number of variables to be included in the multivariable logistic regression model, variables with a *p* value ≤ 0.05 in univariate analysis or those identified in previous studies as potential risk factors were further subjected to the multivariate analysis as described previously (Mei et al. 2010). In brief, we used backward-elimination to examine and determine risk factors for RRD; the entry criteria of 0.05 and removal of 0.10 for the model were set to find the possible risk factors. The statistical significance of partial regression coefficients was analysed with Wald’s chi-square test. Odds ratios (OR) with 95% confidence intervals and the corresponding *p* values were determined for each risk factor. Interactions were not tested. Goodness of fit was determined by the Hosmer-Lemeshow statistic. For multivariable logistic regression and for the analyses of determining which postoperative factors occurred at a higher proportion in the patients who experienced RRD, corrections of *p* value were performed with Benjamini and Hochberg false discovery rate (FDR) method (Benjamini and Hochberg 1995) using an R function p.adjust (R Core Team (2018). R: a language and environment for statistical computing. R Foundation for Statistical Computing, Vienna, Austria. URL https://www.R-project.org/). FDR is the expected proportion of rejected hypotheses that are mistakenly rejected. FDR is a somewhat less conservative/more powerful method for correcting for multiple comparisons than procedures like Bonferroni correction that provide strong control of the family-wise error rate. The FDR is defined as 5% in current study. We used SPSS (Version 12, Chicago, IL 60606, USA) for all statistical analysis.

## Results

**Fig. 1:**
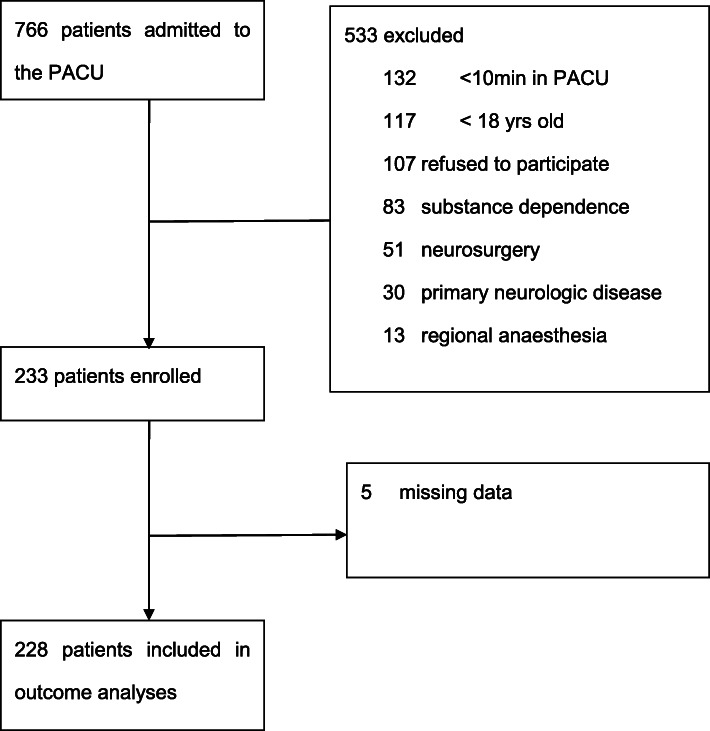
Flow of patients in study cohort

During the study period, 766 patients were admitted to PACU. Exclusion criteria were age < 18 years (*n* = 117), refusal to sign consent form (*n* = 107), operation under regional anaesthesia (*n* = 13), history of substance dependence (including opioid, alcohol, or nicotine) (*n* = 83), neurosurgical procedure (*n* = 51), history of primary neurologic disease (*n* = 30), and admission to PACU with stays of less than 10 min (*n* = 132). Data from five patients were excluded because of incomplete interviews or missing data (Fig. 1). Patients who received general anaesthesia but recovered in locations outside the recovery room (such as ambulance surgery, angiography, endoscopy or electroconvulsive therapy, and cardiac surgery) were not included in this study.

Of the 766 patients admitted to the PACU during the study period, 233 were enrolled in this study, and data from 228 (30%) patients were analysed (Fig. 1). Of these patients, 57 (25%) had delirium, and 171 had no delirium by CAM-ICU. On univariate analysis, the two groups of patients differed with respect to age, ASA-PS, preoperative serum calcium, creatinine, glucose, total or direct bilirubin, serum albumin or total protein, diagnosis of primary disease (malignant or benign), type of surgery (mini-invasive or invasive), location of surgery, maintenance of anaesthesia (inhalation anaesthetic or TIVA), total intraoperative fluid application, total intraoperative body fluid loss, and duration of surgery (Table 1).

**Table 1:** Patient demographic and clinical characteristics

Clinical characteristics	No delirium (*n* = 171)	Delirium (*n* = 57)	*p* value
Age (years)	37 [28–48]	46 [37.5–55]	< 0.001
Age (categories)			0.029
18 ≤ age < 60	162 (76.8%)	49(23.2%)	
Age ≥ 60	9 (52.5%)	8 (47.1%)	
Gender			0.395
Female	101 (77.1%)	30 (22.9%)	
Male	70 (72.2%)	27 (27.8%)	
BMI	21.5 [19.5–24.5]	21.5 [19.4–25.2]	0.713
ASA-PS			< 0.001
I–II	158 (79.4%)	41 (20.6%)	
III–IV	13 (44.8%)	16 (55.2%)	
Primary disease			< 0.001
Benign	156 (80.0%)	39 (20.0%)	
Malignant	15 (45.5%)	18 (54.4%)	
Preoperative LOS	4 [3–6]	5 [3–7]	0.114
Pre-operative blood results			
White blood cell count			0.790
4–10 × 10^9^/L	148 (75.5%)	48 (24.5%)	
< 4 × 10^9^/L	15 (75.0%)	5 (25.0%)	
> 10 × 10^9^/L	8 (66.7%)	4 (33.3%)	
Haemoglobin			0.338
110–150 g/L	135 (77.1%)	40 (22.9%)	
< 110 g/L	25 (65.8%)	13 (34.2%)	
> 150 g/L	11 (73.3%)	4 (26.7%)	
Haematocrit			0.716
37–48%	79 (73.1%)	29 (26.9%)	
< 37%	91 (76.5%)	28 (23.5%)	
> 48%	1 (100.0%)	0 (0.0%)	
Serum sodium			0.052
136–145 mmol/L	126 (73.3%)	46 (26.7%)	
< 136 mmol/L	2 (40.0%)	3 (60.0%)	
> 145 mmol/L	43 (84.3%)	8 (15.7%)	
Serum potassium			0.219
3.5–5.1 mmol/L	142 (77.2%)	42 (22.8%)	
< 3.5 mmol/L	28 (65.1%)	15 (34.9%)	
> 5.1 mmol/L			
Serum chloride			0.993
98–107 mmol/L	146 (74.9%)	49 (25.1%)	
< 98 mmol/L	3 (75.0%)	1 (25.0%)	
> 107 mmol/L	22 (75.9%)	7 (24.1%)	
Serum calcium			0.020
2.16–2.60 mmol/L	155 (77.5%)	45 (22.5%)	
< 2.16 mmol/L	16 (57.1%)	12 (42.9%)	
Serum creatinine			0.041
54-92 μmol/L	78 (67.8%)	37 (32.2%)	
< 54 μmol/L	83 (82.2%)	18 (17.8%)	
> 92 μmol/L	10 (83.3%)	2 (16.7%)	
BUN			0.916
3.2–7.3 mmol/L	143 (74.5%)	49 (25.5%)	
< 3.2 mmol/L	14 (77.8%)	4 (22.2%)	
> 7.3 mmol/L	14 (77.8%)	4 (22.2%)	
Serum total cholesterol			0.882
2.9–5.2 mmol/L	130 (75.6%)	42 (24.4%)	
< 2.9 mmol/L	10 (76.9%)	3 (23.1%)	
> 5.2 mmol/L	31 (72.1%)	12 (27.9%)	
Serum uric acid			0.783
214–488 μmol/L	141 (75.8%)	45 (24.2%)	
< 214 μmol/L	24 (72.7%)	9 (27.3%)	
> 488 μmol/L	6 (66.7%)	3 (33.3%)	
Serum glucose			0.042
3.9–6.4 mmol/L	155 (77.1%)	46 (22.9%)	
< 3.9 mmol/L	10 (71.4%)	4 (28.6%)	
> 6.4 mmol/L	6 (46.2%)	7 (53.8%)	
Elevated serum total or direct bilirubin			0.013
No	154 (77.8%)	44 (22.2%)	
Yes	17 (56.7%)	13 (43.3%)	
Decreased serum albumin or total protein			0.005
No	146 (78.9%)	39 (21.1%)	
Yes	25 (58.1%)	18 (41.9%)	
Elevated hepatic enzymes			0.065
No	142 (72.8%)	53 (27.2%)	
Yes	29 (87.9%)	4 (12.1%)	
Surgical parameters			
Type of surgery			< 0.001
Mini-invasive	100 (88.5%)	13 (11.5%)	
Invasive	71 (61.7%)	44 (38.3%)	
Location of surgery			< 0.004
Head and neck	32 (86.5%)	5 (13.5%)	
Intrathoracic	14 (56.0%)	11 (44.0%)	
Intra-abdominal	49 (65.3%)	26 (34.7%)	
Urogenital	62 (80.5%)	15 (19.5%)	
Musculoskeletal and spinal	9 (100.0%)	0 (0.0%)	
Peripheral	5 (100.0%)	0 (0.0%)	
Maintenance of anaesthesia			< 0.001
TIVA	40 (95.2%)	2 (4.8%)	
Inhalation anaesthetic with isoflurane or sevoflurane	131 (70.4%)	55 (29.6%)	
Preoperative heart rate	75 [66–88]	76 [70.5–92]	0.318
Preoperative systolic BP	117 [108–134]	124 [111–135]	0.103
Preoperative diastolic BP	72 [63–81]	76 [67–87.5]	0.038
Preoperative SpO2 (%)	100 [98–100]	100 [98–100]	0.885
Maximal intraoperative heart rate	90 [80–102]	94[83.5–104.5]	0.201
Minimal intraoperative heart rate	59 [55–65]	57 [52–66]	0.343
Maximal intraoperative systolic BP	131 [120–140]	134 [127–145.5]	0.111
Minimal intraoperative systolic BP	90 [84–96]	89 [82–96]	0.531
Total intraoperative fluid application	1100 [500–1750]	1500[1125–2250]	< 0.001
Total intraoperative body fluid loss	9 [0–400]	300 [45–800]	< 0.001
Duration of surgery			< 0.001
< 120 min	98 (85.2%)	17 (14.8%)	
≥ 120 min	73 (64.6%)	40 (35.4%)	

Data are median [25–75% percentiles] or *n* (%)

On multivariate logistic regression analysis, maintenance of anaesthesia with an inhalation anaesthetic agent (OR = 6.294, 95% CI 1.4–28.8, corrected *p* = 0.030), malignant primary disease (OR = 3.464, 95% CI = 1.396–8.592, corrected *p* = 0.018), ASA-PS III–V (OR = 3.389, 95% CI = 1.401–8.201, corrected *p* = 0.018), elevated serum total or direct bilirubin (OR = 2.535, 95% CI = 1.006–6.388, corrected *p* = 0.049), and invasive surgery (OR = 2.431, 95% CI = 1.103–5.357, corrected *p* = 0.035) were identified as independent risk factors for RRD in non-cardiovascular surgery patients (Table 2). The model fitted the data well (*p* = 0.68 by the Hosmer-Lemeshow test).

**Table 2:** Independent risk factors for RRD after general anaesthesia in elective non-cardiovascular surgery patients

	Regression coefficient (SE)	Odds ratio	95.0% CI for odds ratio		Corrected *p*
			Lower	Upper	
Maintenance of anaesthesia with inhalation anaesthetic	1.840 (0.775)	6.294	1.377	28.759	0.030
Malignant primary disease	1.242 (0.464)	3.464	1.396	8.592	0.018
ASA-PS III-V	1.221 (0.451)	3.389	1.401	8.201	0.018
Elevated serum total or direct bilirubin	0.930 (0.472)	2.535	1.006	6.388	0.049
Invasive surgery	0.888 (0.403)	2.431	1.103	5.357	0.035

Other variables included in the model were older age, gender, BMI, location of surgery, decreased serum albumin or total protein, preoperative serum creatinine, preoperative serum calcium, preoperative serum glucose, intraoperative fluid application, total intraoperative body fluid loss, and duration of surgery. *p* values were corrected with Benjamini and Hochberg false discovery rate method

Patients with RRD had significantly higher maximal SBP (141 mmHg [131–151] versus 132 mmHg [122–143], corrected *p* = 0.005) and higher minimal SBP (120 mmHg [115–132] versus 115 [106–125], corrected *p* = 0.002), had higher total healthcare costs (31,428 yuan [17,872–43,674] versus 16,555 yuan [12,618–27,788], corrected *p* = 0.002), had a longer median length of hospital stay (17 days [12–23.5] versus 11 days [9–17], corrected *p* = 0.002), and longer postoperative stay (11 days [7–15] versus 7 days [5–10], corrected *p* = 0.002]), and longer PACU stay (34 min [25–43.5] versus 29 min [23–37], corrected *p* = 0.030) (Table 3).

**Table 3:** PACU events, LOS, and healthcare costs

	No delirium (*n* = 171)	Delirium (*n* = 57)	Corrected *p* value
Maximal heart rate in PACU (bpm)	90 [80–100]	86 [77–98]	0.293
Maximal SBP in PACU (mmHg)	132 [122–143]	141 [131–151]	0.005
Minimal SBP in PACU (mmHg)	115 [106–125]	120 [115–132]	0.002
Mean SpO2 (%) in PACU	99 [97–100]	98 [96–100]	0.340
PACU LOS (min)	29 [23–37]	34 [25–43.5]	0.030
Total hospital LOS (days)	11.0 [9.0–17.0]	17.0 [12.0–23.5]	0.002
Postoperative LOS (days)	7.0 [5.0–10.0]	11.0 [7.0–15.0]	0.002
Healthcare costs per day (yuan)	1459 [1217–1966]	1696 [1285–2106]	0.064
Total healthcare costs(yuan)	16,555 [12,618–27,788]	31,428 [17,872–43,674]	0.002

Data are median [25–75% percentiles]; Bonferroni corrected *p* value is 0.006. *p* values were corrected with Benjamini and Hochberg false discovery rate method

## Discussion

According to CAM-ICU flowsheet, one quarter of the patients in our study experienced RRD after general anaesthesia for elective non-cardiovascular surgery. Our results are similar to previous studies (Card et al. 2015; Fields et al. 2018). Using the Confusion Assessment Method (CAM) score, Sharma et al. reported 45% of elderly patients have RRD after hip-fracture repair surgery (Sharma et al. 2005). Using the Riker Sedation–Agitation Scale, Lepouse et al. reported a delirium rate of 4.7% in adults in the PACU (Lepouse et al. 2006). Radtke et al. reported that delirium in the recovery room was seen in 21 patients (14%) with the Diagnostic and Statistical Manual of Mental Disorders -IV (DSM-IV) criteria, in 11 patients (7%) with the CAM score, in four patients (3%) with the Delirium Detection Score (DDS), and in 37 patients (24%) with the Nursing Delirium Screening Scale (Nu-DESC) in the same patient population (Radtke et al. 2008). The prevalence rate for delirium is greatly affected by the diagnostic formulation used (Card et al. 2015; Fields et al. 2018; Voyer et al. 2009). The definition of the outcome measure, the length of the post-operative observation period, and the patient population also cause differences in observed delirium rates. Exclusion criteria may also affect delirium rates, as cardiac surgery and neurosurgery are major contributors to postoperative delirium (Oh et al. 2008; Rudolph et al. 2009). Development of a widely accepted scale for detecting RRD in the postoperative setting would improve the timely diagnosis and management of RRD.

In the present study, a greater proportion of patients who received isoflurane or sevoflurane for maintenance anaesthesia experienced RRD than patients who received TIVA. Multivariate logistic regression analysis confirmed that isoflurane or sevoflurane for maintenance anaesthesia was the strongest risk factor for RRD. Previous studies have shown that inhalation anaesthetics such as isoflurane and sevoflurane are associated with postoperative delirium during recovery, particularly in young children or elderly patients (Aono et al. 1997). Very few studies have compared the incidence of delirium in adults anaesthetised with inhalation anaesthetics and those anaesthetised with propofol (Lepouse et al. 2006; Nishikawa et al. 2004). Lepouse et al. found more agitated patients had been anaesthetised with inhalation anaesthetics (62%) than with propofol (37%), but multivariate analysis did not confirm this result (Lepouse et al. 2006). Several studies have demonstrated a protective effect of propofol on postoperative delirium in children (Aouad et al. 2007), although this is controversial (Konig et al. 2009). Old rats are more profoundly influenced than young adult rats by isoflurane anaesthesia with regard to reductions in acetylcholine release and stress responses (Jansson et al. 2004). In addition, isoflurane-induced beta-amyloid protein oligomerization and apoptosis may contribute to the risk of postoperative cognitive dysfunction (Xie et al. 2006). Inhalation anaesthetic agents may thus increase the odds of postoperative delirium in specific populations. Testing this hypothesis in a well-designed prospective study may give further evidence in this direction.

Our data showed that patients undergoing surgery for malignant disease had higher proportion of RRD than patients with benign disease, and our multivariate logistic regression analysis confirmed malignant primary disease as an independent risk factor for RRD. Delirium occurs in 26 to 44% of cancer patients (Centeno et al. 2004), and 74% of patients with advanced cancer experience an episode of delirium (Bruera et al. 2009). Structural brain lesions and toxic or metabolic encephalopathy are thought to be causes of delirium in cancer patients (Doriath et al. 2007). Our data suggest that cancer patients undergoing surgery are at increased odds of RRD. Whether interventions for the prevention of delirium in cancer patients result in better short- or long-term outcomes after surgery are unknown. Prevention of delirium, however, is desirable for cancer patients and their anaesthetists (Siddiqi et al. 2007).

Univariate analyses showed a higher proportion of patients with RRD were ASA-PS III–V. Multivariate logistic regression analyses confirmed higher ASA-PS to be an independent risk factor for RRD. Clinical studies of such differences have produced conflicting results. Higher ASA-PS was identified as a risk factor after abdominal surgery in univariate but not in multivariate analysis in a previous study with a small patient population (Koebrugge et al. 2009). Illness severity was also associated with risk of delirium in a prospective study in hospitalised elderly (Francis et al. 1990). Moreover, delirium was the most common neuropsychiatric complication experienced by patients with advanced illness, occurring in up to 85% of patients in the last weeks of life (Breitbart and Alici 2008). Consistent with our study, Zakriya et al. reported ASA physical status > II to be one of three significant predictors of postoperative delirium in geriatric patients (OR = 11.3, 95% CI 2.6–49.2, *p* < 0.001) (Zakriya et al. 2002).

Elevated serum total or direct bilirubin was more frequent in the RRD group, and multivariate analysis confirmed elevated total or direct serum bilirubin as an independent risk factor for RRD. Literature examining the relationship between bilirubin and delirium is limited. Dubois et al. demonstrated that abnormal bilirubin levels were associated with delirium in the intensive care unit (Dubois et al. 2001). Direct bilirubin is also assumed to play a role in the pathogenesis of hepatic encephalopathy (Muller et al. 1994). Due to the small number of patients with elevated bilirubin in our population (*n* = 30), we recommend caution in interpreting this result.

We showed that invasive surgery was an independent risk factor for RRD, in accordance with many previous studies. Low operative stress procedures such as cataract surgery resulted in delirium in 4.4% cases (Milstein et al. 2002), whereas higher stress procedures such as acute hip fracture surgery resulted in delirium in 40% of cases (Marcantonio et al. 2002). Shiiba et al. reported that postoperative delirium was associated with extensive surgery for oral carcinoma (Shiiba et al. 2009). The degree of operative stress may be one of factors affecting RRD. Mini-invasive endoscopic surgery may prevent RRD in high-risk patients, but a proper randomised trial would be required to test this hypothesis.

Several studies demonstrated that older age (Koebrugge et al. 2009); abnormal preoperative sodium, potassium, or glucose levels (Galanakis et al. 2001; Marcantonio et al. 1994); diabetes mellitus (Gao et al. 2008); haemoglobin < 100 g/L (Gao et al. 2008); hypoalbuminemia (Robinson et al. 2009); longer operation time (Yildizeli et al. 2005); massive blood transfusion (Katznelson et al. 2009b); abnormal postoperative sodium, potassium, or glucose levels (Yildizeli et al. 2005); and postoperative haematocrit < 30% (Marcantonio et al. 1998) were important in influencing postoperative delirium. In our study, older age, decreased preoperative serum calcium, elevated preoperative serum glucose, decreased preoperative serum total protein or albumin, location of surgery, total intraoperative body fluid loss and intraoperative fluid application, and duration of surgery were significant in univariate but not multivariate analyses. While both young and old age have been associated with delirium, our cohort had only a few patients ≧ 60-year old (*n* = 17), potentially negating the impact of age on delirium in our study. Difference in study design, study population, and the definition of outcome parameters may account for the variance. Some of these parameters seem to play a role, however, and should be included in future prospective studies.

We did not include some variables reported to influence postoperative delirium such as a history of central nervous system disorder (Gao et al. 2008), pre-existing dementia (Robinson et al. 2009), preoperative depression (Katznelson et al. 2009b), preoperative alcohol use (Williams-Russo et al. 1992), postoperative pain (Oh et al. 2008), and preoperative medication such as beta-blockers (Katznelson et al. 2009a) in our analyses. Many of these variables are not included in our routine clinical data with enough reliability, and we excluded patients with central nervous system disease. We are thus unable to report on the relative contribution of these factors in our patients.

Delirium in the surgical/trauma ICU cohort is associated with more days of mechanical ventilation and more days in ICU and hospital (Lat et al. 2009). Elderly subjects with postoperative delirium have a greater hospital LOS, are more likely to be institutionalised after discharge, and have a higher 6-month mortality than those without delirium (Robinson et al. 2009). After elective surgery in older adults, delirium significantly prolonged hospital LOS (Gleason et al. 2015). Postoperative delirium after liver transplantation is associated with increased intensive care unit and hospital LOS (Beckmann et al. 2017; Bhattacharya et al. 2017). As with former studies, our univariate analyses demonstrated that patients with RRD stayed longer in PACU and had longer hospital and postoperative stays. Franco et al. demonstrated that postoperative delirium is an extremely costly disorder in patients undergoing elective surgery (Franco et al. 2001). After spine surgery in older adults, the development of delirium was independently associated with higher hospital charges (Brown et al. 2016). Patients with postoperative delirium after urologic cancer surgeries experienced worse outcomes, prolonged LOS, and increased admission costs (Ha et al. 2018). Consistent with previous study, our study demonstrated that patients with RRD had higher total healthcare costs. It has been reported that intraoperative hypotension was not associated with the occurrence of delirium after cardiac surgery (Wesselink et al. 2015), whereas a recent study demonstrated that a progressive decrease in mean arterial blood pressure during surgery was associated with the increased odds of developing postoperative delirium (Radinovic et al. 2019). In elderly hip fracture patients, both very high and very low levels of mean arterial blood pressure were associated with significantly increased risk of postoperative delirium (Wang et al. 2015). In addition, increased blood pressure fluctuation was predictive of early postoperative delirium after non-cardiac surgery (Hirsch et al. 2015). In consistent with previous study, our data demonstrated that patients with RRD had higher SBP in PACU. These may imply that blood pressure level may be associated with delirium in a context-dependent nature.

Our study has several limitations. Due to the observational design, a causal link between the proposed risk factors and RRD cannot be inferred. Choosing exclusion criteria to reduce the possibility of confounding factors may have influenced the results, as excluding patients undergoing cardiac surgery, neurosurgery, as well as patients with history of substance dependence (including opioid, alcohol, or nicotine), may have reduced the proportion of RRD. The patient group we investigated was not a consecutive sample so selection bias is possible. In addition, the time of the study was performed in 2010. However, the patients in this study were representative of the type of patients treated in our hospital. We believe that the data in this study is still valuable and could provide reference for clinical practice.

## Conclusion

This is the first study concerning recovery room delirium in Chinese populations. One quarter of elective non-cardiovascular surgery patients experienced RRD after general anaesthesia. On multivariate analysis, maintenance of anaesthesia with inhalation agents (sevoflurane or isoflurane), malignant disease, ASA-PS III-V, elevated serum total or direct bilirubin, and invasive surgery were identified as risk factors for RRD in these patients. Our results show delirium is a major complication in the PACU that is associated with higher healthcare costs and increased post-operative LOS. Identifying patients at risk of RRD after non-cardiovascular surgery should enable earlier recognition and intervention in postoperative delirium, which may lead to improved short- and long-term patient outcomes (Siddiqi et al. 2007).

## Data Availability

The datasets used and/or analysed during the current study are available from the corresponding author on reasonable request.

## Abbreviations

ASA-PS: American Society of Anaesthesiologists Physical Status
BUN: Blood urea nitrogen
CAM: Confusion Assessment Method
CAM-ICU: Confusion Assessment Method for the Intensive Care Unit
DDS: Delirium Detection Score
DSM-IV: Diagnostic and Statistical Manual of Mental Disorders-IV
LOS: Length of stay
Nu-DESC: Nursing Delirium Screening Scale
PACU: Post-anaesthesia care unit
RRD: Recovery room delirium
SBP: Systolic blood pressure
TIVA: Total intravenous anaesthesia
